# PARENTHOOD AND SLEEP IN A POPULATION-BASED SAMPLE OF OLDER ADULTS

**DOI:** 10.1093/geroni/igae098.3523

**Published:** 2024-12-31

**Authors:** Jacquelyn Duckett, Rodlescia Sneed

**Affiliations:** Wayne State University, Detroit, Michigan, United States; Wayne State University, Detroit, Michigan, United States

## Abstract

Numerous studies have examined the association between parenthood and health-related outcomes in young and middle-aged adults; however, there has been less inquiry into how parenthood is associated with health-related outcomes among adults aged >50. Further, few studies have explored how various aspects of the parenthood experience (e.g., number of children, geographic proximity, frequency/nature of contact, relationship quality) are differentially associated with health-related outcomes. The current study aimed to evaluate associations between parenthood and sleep, a critical factor in maintaining health among adults aged >50. We addressed our research questions via secondary analysis of data from the 2016 and recent waves of the Health and Retirement Study, a nationally representative population-based study of community-dwelling U.S. adults aged >50 (n= 4,201). Both parenthood measures and sleep outcomes were measured via self-report. We used multiple linear regression to investigate how various aspects of parenthood were differentially related to sleep outcomes, controlling for demographic characteristics, chronic health conditions, and baseline sleep scores. We observed that more positive interactions and fewer negative interactions with children were associated with better sleep. None of our other parenthood measures were associated with sleep outcomes. Our findings suggest that interaction quality, but not other aspects of the parent-child relationship, is important for the sleep hygiene of older adults.

